# Stroke Mortality Attributable to Low Fruit Intake in China: A Joinpoint and Age-Period-Cohort Analysis

**DOI:** 10.3389/fnins.2020.552113

**Published:** 2020-12-01

**Authors:** Lisha Luo, Junfeng Jiang, Chuanhua Yu, Mingjuan Zhao, Yunyun Wang, Quanlei Li, Yinghui Jin

**Affiliations:** ^1^Center for Evidence-Based and Translational Medicine, Zhongnan Hospital of Wuhan University, Wuhan, China; ^2^Center for Evidence-Based and Translational Medicine, Wuhan University, Wuhan, China; ^3^School of Health Sciences, Wuhan University, Wuhan, China; ^4^School of Nursing, Johns Hopkins University, Baltimore, MD, United States

**Keywords:** stroke, low fruit intake, age–period–cohort analysis, joinpoint analysis, relative risk

## Abstract

Stroke is the first leading cause of death in China, and low fruit intake is suggested to be one of the most important risk factors for stroke mortality. However, the trends of stroke mortality attributable to low fruit intake remain unclear in China. Therefore, this study aimed to investigate the long-term trends of stroke mortality attributable to low fruit intake by sex in China during 1990–2017. Data were obtained from the Global Burden of Disease 2017 study; the annual percentage change (APC) and average annual percentage change (AAPC) were estimated by joinpoint regression analysis, and the net age, period, and cohort effects were estimated using the age–period–cohort model with an intrinsic estimator algorithm (APC-IE). The crude mortality rates (CMRs) increased for males and decreased for females from 1990 to 2017. The age-standardized mortality rates (ASMRs) for both males and females showed consecutive significant declines from 1990 to 2017. By APC analysis, substantially increasing age effects were presented from 25 to 79 years for both sexes. The independent period and cohort effects progressively decreased during the entire period for both sexes, with a faster decrease for females than for males. Males and elder groups were the high-risk population for stroke mortality caused by low fruit intake. Although the mortality risk showed a decreasing trend, the fruit intake was still low for the Chinese population. Therefore, effective strategies and global awareness are essential to improve the current situation of low fruit intake, thereby preventing and reducing the stroke mortality risk caused by low fruit intake in China.

## Introduction

As one of the non-communicable diseases (NCDs), stroke has become the second leading cause of death globally, which has been identified by the World Health Organization (WHO) and the United Nations as one of the priorities for actions to reduce the burden of NCDs (Feigin et al., 2015; GBD Collaborators, 2019). As the Global Burden of Disease 2017 (GBD 2017 Disease and Injury Incidence and Prevalence Collaborators, 2018) reported, there were approximately 6.17 million deaths and 132.05 million disability-adjusted life years (DALYs) caused by stroke in 2017, and the mortality decreased to 80.72 per 10,000 in 2017 (GBD Collaborative Network Network, 2018). Stroke had always been the first fatal disease in China, and the mortality rate increased from 105.93 per 10,000 in 1990 to 149.37 per 10,000 in 2017, which has brought a massive health and economic burden to society (Zhou et al., 2016; GBD Collaborative Network Network, 2018). It is reported that stroke is caused by various risk factors; therefore, it is urgent to identify the controllable risk factors that lead to the higher level in the disease burden of stroke.

The WHO has recommended >400 g of fruit and vegetable intake per day for a population (WHO, 2019), and previous epidemiology studies concluded that low fruit intake was associated with a variety of NCDs, like diabetes, kidney disease, neoplasms, and cardiovascular disease (Li et al., 2014; Yu et al., 2014; GBD Collaborators, 2018; Mottaghi et al., 2018). The China Kadoorie Biobank Study, a large national prospective cohort study, indicated that a higher level of fresh fruit intake was associated with lower blood pressure and lower risk of cardiovascular disease (Du et al., 2016; Tian et al., 2017). A meta-analysis concluded that a 200-g per day increase in fruit intake would decrease 32% risk of stroke (Hu et al., 2014). The United Kingdom women’s cohort study indicated that each 80-g per day intake of fruit could reduce 6–7% mortality risk of cardiovascular and coronary heart disease (Lai et al., 2015). According to the GBD 2017, low fruit intake was the fourth highest-ranking risk factor for stroke globally, and there were about 14.77 per 10,000 of stroke deaths caused by low fruit intake in 2017. In China, low fruit intake has become the third independent risk factor for stroke, and the disease burden was more serious. In 2017, there were approximately 0.4 million stroke deaths caused by low fruit intake in China, accounting for 35% of the world’s deaths (GBD Collaborative Network Network, 2018). The age-standardized stroke mortality attributable to low fruit intake in 2017 in China (21.34 per 10,000) was much higher than that in the United States (3.71 per 10,000) (GBD Collaborative Network Network, 2018). Therefore, it was significant to estimate the stroke burden attributable to low fruit intake in China.

Most previous studies in China focused on the association between a combination of fruit and vegetable intake and different diseases, not fruit alone (Du et al., 2016). Furthermore, there was no comprehensive study to explore the long-term trends of stroke mortality attributable to low fruit intake between different age groups and gender, and to analyze these trends from age, period, and cohort dimensions. As far as we know, analyzing the long-term trends of stroke mortality attributable to low fruit intake was of great significance in identifying its influencing factors, especially in different social backgrounds. Therefore, this study aimed to investigate the independent effects of age, period, and cohort on stroke mortality attributable to low fruit intake during 1990 to 2017 in China, compare these effects by sex using data from GBD 2017, and discuss the possible influencing factors. Our findings would provide an epidemiological basis for the prevention of stroke, so as to reduce the disease burden of stroke due to controllable risk factors.

## Materials and Methods

### Data Source

Data were obtained from GBD 2017, which provided a comprehensive assessment of cause-specific mortality for 354 causes of death and 84 risk factors or clusters in 195 countries and territories from 1990 to 2017 (GBD 2017 Disease and Injury Incidence and Prevalence Collaborators, 2018). The main characteristics of the data used in this study are shown in Table 1. The original data for estimating low fruit intake in China were collected from China chronic disease and risk factor surveillance, fresh foods market statistics, China National Nutrition Survey, and other scientific literatures (GBD Collaborators, 2017). Data source on stroke mortality included Cause of Death Reporting System of the Chinese Center for Disease Control and Prevention, Disease Surveillance Points, and Maternal and Child Surveillance System, which covered at least 323.8 million population in China (Wang Z. et al., 2017; GBD 2017 Disease and Injury Incidence and Prevalence Collaborators, 2018; Cao et al., 2019). The reliability and representativeness of China part in GBD 2017 have been officially recognized, and many researches using the data have been published by the Chinese Center for Disease Control and Prevention in the Lancet (Zhou et al., 2019). Stroke was defined based on the 9th and 10th version of the International Classification of Disease (Krishnamurthi et al., 2015; GBD Collaborators, 2018). GBD 2017 used a comparative risk assessment framework to estimate the stroke mortality attributable to low fruit intake, which was equal to stroke mortality multiplied by the population attributable fraction (PAF) for low fruit intake for each age group, sex, and year (Feigin et al., 2016; GBD Collaborators, 2018). The PAF was the proportion of stroke mortality reduced if low fruit intake was reduced to the level of theoretical minimum risk exposure level (average daily consumption of fruit 200–300 g). Age-standardized rates were based on the GBD 2017 global age-standard population (Zhou et al., 2019).

**TABLE 1:** The main characteristics of the data used in this study.

**Category**	**Content**
Source	GBD 2017
Year	1990–2017
Country	China
Sex	Males and females
Age	All ages, 25–29, 30–34, 35–39, 40–44, 45–49, 50–54, 55–59, 60–64, 65–69, 70–74, 75–79, 80–84, Age-standardized
Disease	Stroke
Risk factor	Low fruit intake
Index	Mortality (per 10,000)

### Statistical Analysis

In this study, the joinpoint analysis was used to estimate the time trends of stroke mortality attributable to low fruit intake during 1990 to 2017, which could divide the long-term trends of mortality into segments and identify the trends with statistical significance in different segments (Kim et al., 2000; Ilic and Ilic, 2017). The mortality rate in this study followed a Poisson distribution, so a logarithmic transformation was carried out. The annual percent change (APC), average annual percent change (AAPC), and corresponding 95% confidence interval (CI) were calculated using the joinpoint analysis in this study to estimate the stroke mortality attributable to low fruit intake by sex during 1990–2017 (Clegg et al., 2009; Rea et al., 2017).

The age–period–cohort (APC) analysis was conducted in this study to explore the cumulative health risks since birth that were included in the mortality. The APC model could estimate the net age, period, and cohort effects on stroke mortality attributable to low fruit intake simultaneously. The specific equation was exhibited as follows:

$$Y_{i}=\mu +\alpha \times {\text{age}}_{i}+\beta \times {\text{period}}_{i}+\gamma \times {\text{cohort}}_{i}+\varepsilon$$

where *Y*_*i*_ denotes the outcome-stroke mortality attributable to low fruit intake for groups *j*, α, β, and γ denoted the coefficient of age, period, and cohort of the APC model, respectively, and μ denoted the intercept of the model, and ε denoted the residual of the APC model (Yang et al., 2004; Harding, 2009; Luo et al., 2017).

However, there was an exact linear relationship among age, period, and cohort. It was impossible to get the net effects for these three different temporal dimensions. To address this identification problem, the intrinsic estimator (IE) algorithm was used to solve the APC problem. The APC-IE model has been proved to be unbiased, estimable, and valid in solving the identification problem (Yang, 2008; Yang et al., 2008). To conduct the APC-IE model, stroke mortality attributable to low fruit intake was recoded into consecutive 5-year periods from 1990 to 2017 and successive 5-year age groups from 25–29 to 80–84 years. Because the population over 85 years was recorded as one group in GBD 2017, the mortality in this group was excluded in our study. Those of 1990 and 1991 were excluded from the APC model because they were not enough for 5-year periods. To intuitively interpret the trends of age, period, and cohort effects on stroke mortality attributable to low fruit intake, we calculated the relative risk (RR) based on estimated coefficient. The first group of 25–29 years, 1992 time period, and 1908–1912 cohort were defined as the reference groups. The RR was the exponent of the difference in estimated coefficient between the other groups and the reference groups.

In the present study, the joinpoint regression software version 4.5.0 was used to run the joinpoint analysis. The APC-IE model was carried out using the Stata 12.0 (StataCorp, College Station, TX, United States), and Deviance, Akaike Information Criterion (AIC), and Bayesian Information Criterion (BIC) were reported to evaluate the goodness of model fit.

## Results

**FIGURE 1:**
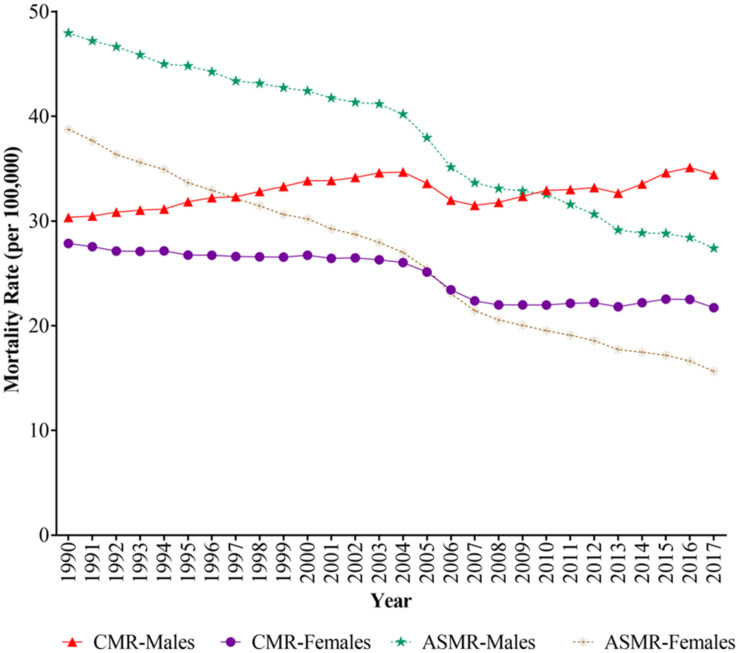
The trends of crude mortality rates (CMRs) and age-standardized mortality rates (ASMRs) for stroke mortality attributable to low fruit intake during 1990–2017.

The trends of stroke mortality attributable to low fruit intake for males and females during 1990 to 2017 are presented in Figure 1, the APC and AAPC by joinpoint analysis are displayed in Table 2. We can observe that crude mortality rates (CMRs) and age-standardized mortality rates (ASMRs) of stroke attributable to low fruit intake for males were both higher than that for females. The CMRs for males increased by 1.0% per year from 1990 to 2004, then decreased by 3.3% per year from 2004 to 2007 and increased by 1.0% per year during 2007–2017. By contrast, females’ CMRs decreased from 1990 to 2007, then remained at stable level from 2007 to 2017. The ASMRs decreased by 2.1 and 3.4% per year for males and females during 1990–2017, respectively.

**TABLE 2:** The APCs and AAPCs of stroke mortality attributable to low fruit intake by sex in China, 1990–2017.

**Segments**	**Crude mortality rates (CMRs)**		**Age-standardized mortality rates (ASMRs)**	
	**Year**	**APC (95% CI)**	**Year**	**APC (95% CI)**
**Males**				
Trend 1	1990–2004	1.0 (0.9, 1.2)*	1990–2004	−1.2 (−1.3, −1.1)*
Trend 2	2004–2007	−3.3 (−6.1, −0.6)*	2004–2007	−6.1 (−8.0, −4.1)*
Trend 3	2007–2017	1.0 (0.8, 1.3)*	2007–2010	−0.8 (−2.9, 1.5)
Trend 4			2010–2013	−3.4 (−5.6, −1.2)*
Trend 5			2013–2017	−1.5 (−2.2, −0.7)*
AAPC (95% CI)	1990–2017	0.3 (0.2, 0.5)*	1990–2017	−2.1 (−2.3, −1.9)*
**Females**				
Trend 1	1990–2004	−0.3 (−0.5, −0.2)*	1990–2004	−2.4 (−2.5, −2.3)*
Trend 2	2004–2007	−5.4 (−8.0, −2.7)*	2004–2007	−7.7 (−9.9, −5.5)*
Trend 3	2007–2017	0.0 (−0.2, 0.2)	2007–2017	−2.9 (−3.1, −2.7)*
AAPC (95% CI)	1990–2017	−3.4 (−3.6, −3.2)*	1990–2017	−3.4 (−3.6, −3.2)*

*The value was statistically different from zero (*P* < 0.05). APCs, annual percent changes; AAPCs, average annual percent changes.

**FIGURE 2:**
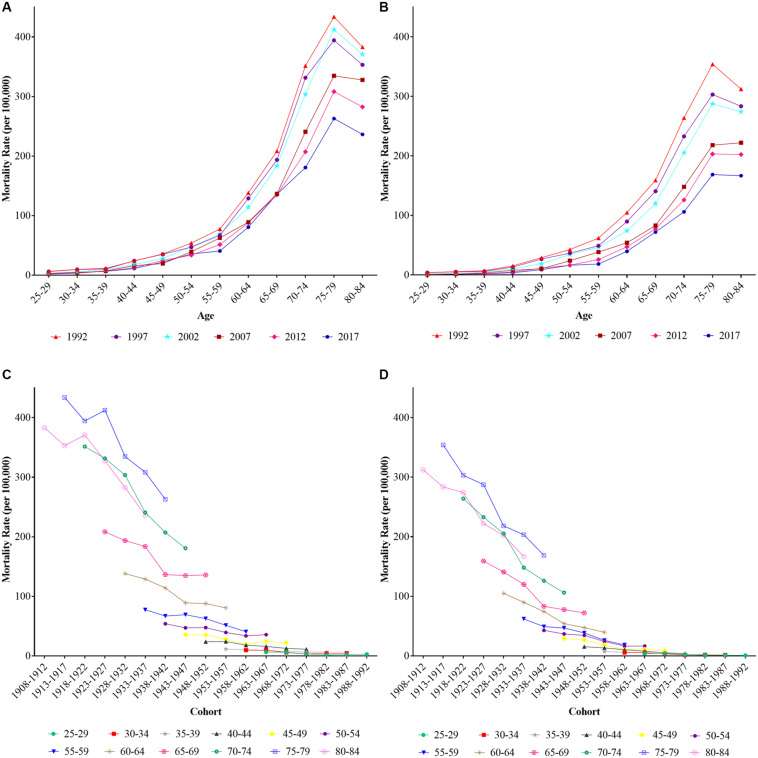
The common long-term trends of age-specific and cohort-based variation of stroke mortality attributable to low fruit intake in China during 1990–2017. **(A,B)** Age-specific mortality for males and females and **(C,D)** cohort-based variation of mortality for males and females, respectively.

The description of age-specific and cohort-based variation of stroke mortality attributable to low fruit intake for both sexes from 1992 to 2017 are depicted in Figure 2. The mortality showed a similar exponential increase with age for both males and females, and the rate accelerated before the group aged 50–54 years, peaked at group aged 75–79 years, then decreased thereafter. The age-specific mortality rates in all age groups for males were higher than those for females. The mortality rates for all age groups decreased across birth cohorts, and these tendencies were more obvious in elder groups. Because this cohort variation might be confounded by age and period, it was impossible to evaluate the net cohort effect. Therefore, APC-IE was used to address this limitation.

**FIGURE 3:**
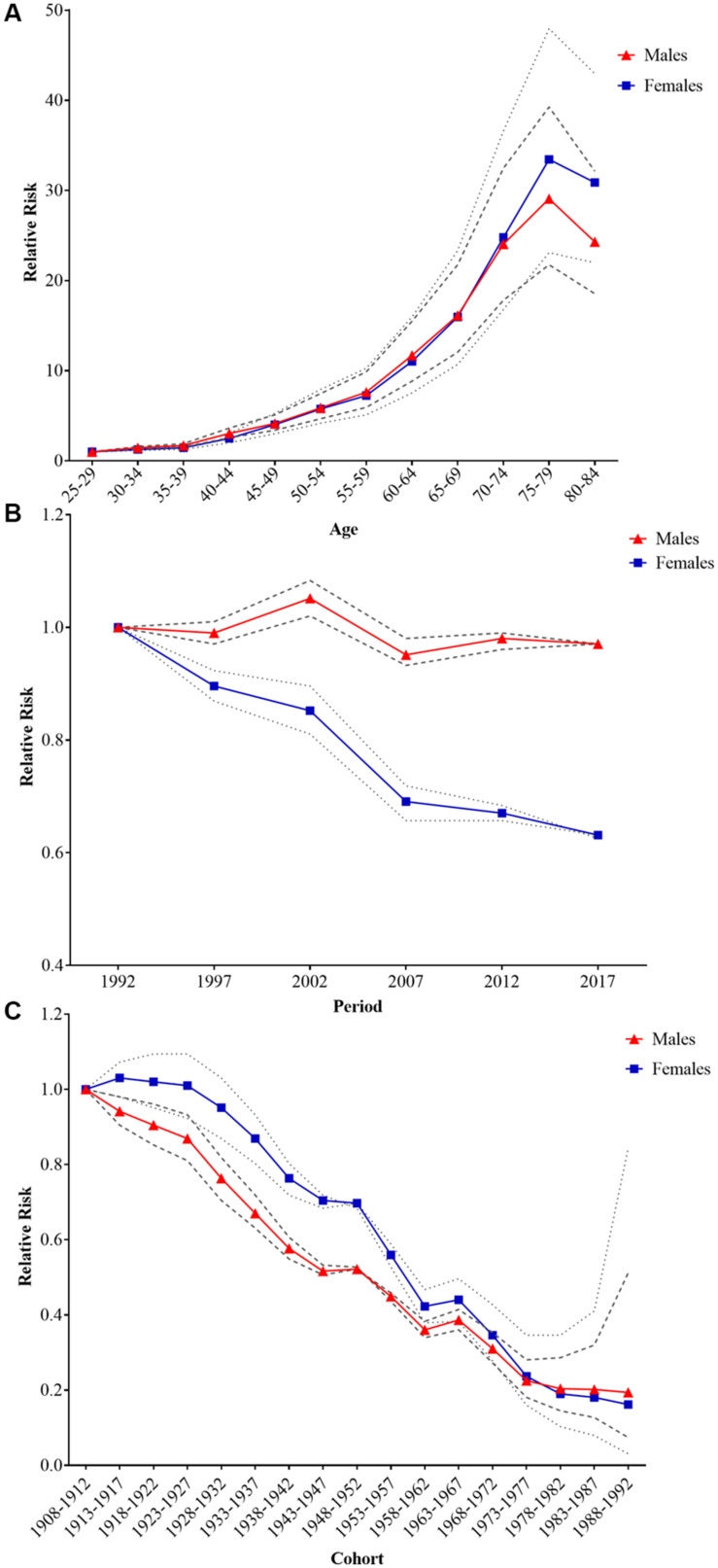
The age, period, and cohort effects on stroke mortality attributable to low fruit intake in China by age–period–cohort analysis. **(A–C)** Age, period, and cohort effects, respectively, and the thick gray line represents the 95% confidence interval (CI) for males, and the thin gray line represents the 95% CI for females.

The RR of age, period, and cohort effects of stroke mortality attributable to low fruit intake for both sexes estimated by APC-IE analysis are shown in Figure 3 and Supplementary Table 1 presented the specific coefficients and standard errors of the three effects. After controlling for period and cohort effects, a significant age effect on stroke mortality attributable to low fruit intake was yielded. The mortality risk increased with advancing age for both sexes until 75–79 years, then decreased thereafter, and the RR in the group aged 75–79 were 31.50 and 38.09 times greater than those in the group aged 25–29 for males and females, respectively. The period effect of the mortality risk for both males and females showed a downward trend between 1992 and 2017, with a faster decline for females than for males. Compared with the period effect in 1990, the RR of mortality in 2017 was 0.94 and 0.59 for males and females, respectively. The cohort effect for both sexes showed significant downward trends, and the earlier birth cohorts had experienced higher mortality risk, while the lower mortality risks were found in the more recent birth cohorts.

## Discussion

Many previous studies have proved that multiple dietary factors can influence the occurrence and development of stroke (Feigin et al., 2016). Low fruit intake is the second independent dietary risk factor for stroke after a high sodium intake; therefore, the disease burden of stroke caused by it cannot be ignored (GBD Collaborators, 2018). The main potential mechanisms for fruit decreasing stroke mortality risk are that some fruits (extracts) have antioxidant and free radical scavenging functions, such as grapes, blueberries, pomegranates, apples, hawthorns, and avocados (Brasil et al., 2014; Zhao et al., 2017). A previous study revealed that the mechanisms by which fruit protects the cardiovascular system included protecting vascular endothelial function, regulating lipid metabolism, modulating blood pressure, inhibiting platelet function, alleviating ischemia/reperfusion injury, suppressing thrombosis, reducing oxidative stress, and attenuating inflammation. Consequently, increasing people’s fruit intake should be considered by policymakers as one of the most recommended effective strategies in stroke prevention (Zhao et al., 2017).

Our study aimed to analyze the long-term trends of stroke mortality attributable to low fruit intake and examined the net age, period, and cohort effects separately by sex in China during 1990 to 2017. We concluded that males had higher stroke mortality rates attributable to low fruit intake than females. During 1990–2017, the CMRs increased for males, while they decreased for females, and ASMRs showed similar downward patterns for both sexes. The sex disparities of stroke mortality attributable to low fruit intake indicated that males were more vulnerable to low fruit intake, which was mainly associated with their high baseline mortality of stroke and low-level fruit consumption. Because of the higher exposure to many risk factors of stroke, the baseline stroke mortality for males was higher than that for females (Wang Z. et al., 2017). In addition, there was a significant difference in fruit consumption between males and females; the average daily fruit intake was 38.1 g for male adults and 49.4 g for female adults in 2009, which was mainly attributed to their biological disparities (Zhang, 2013). Compared to males, females had stronger health awareness and were more likely to receive health-related information and changed their unhealthy lifestyles (Barker-Collo et al., 2015). Because low fruit intake was a controllable risk factor, therefore, we should take measures to strengthen the health awareness of population, especially for males, so as to help them live a healthier life.

Many previous studies confirmed that age was an independent and important risk factor for stroke, and the death and disability caused by stroke varied across different age groups (Wang J. et al., 2017; Cao et al., 2019). In the present study, we can see that the stroke mortality attributable to low fruit intake increased with advancing age; the elder groups suffered higher mortality risks, which were consistent with the age trends of stroke mortality (Zhou et al., 2016; Li et al., 2017). One study using data from Chinese Health Statistics Yearbooks indicated that the higher stroke mortality risks were distributed in elder groups (Li et al., 2017). Stroke mortality attributable to low fruit intake was the product of stroke mortality and PAF of low fruit intake; therefore, baseline mortality rates had significant impacts on outcomes. The age effect might be influenced by biological factors; as far as we know, older adults had poor immune functions and were more likely exposed to various risk factors of stroke. Also, they tended to have lower consumption of fruit than young people because of the need to control blood glucose (He et al., 2016). In addition, our study indicated that the stroke mortality due to low fruit intake in the 80–84 age group was lower than that in the 75–79 age group, which was consistent with previous studies (Wang Z. et al., 2017; Cao et al., 2019). According to the GBD data, we found that the PAF of low fruit intake decreased with age, and the 80–84 age group had the lowest value, as this age group had the lowest consumption of fruits (GBD Collaborators, 2018). Because the stroke mortality and population attribution fraction of low fruit intake both showed downward trends in the group aged 80–84 years, the attributable mortality risk also decreased in this group.

Period effects indicated that the stroke mortality attributable to low fruit intake decreased from 1990 to 2017 for both sexes, and females had a more obviously decreasing trend than males, as mentioned above, because females were more likely to receive health-related information and execute health behaviors. The China Health and Nutrition Survey revealed that the daily fruit intake increased from 8.0 g/day in 1991 to 38.1 g/day in 2009 for males and 10.3 g/day in 1991 to 49.4 g/day in 2009 for females (Zhang, 2013; Xiao et al., 2015), which partly explains the findings of the present study. There are many reasons for the upward trend in fruit consumption; for example, the living standard of population had gradually improved with the rapid development of economy, and people’s health awareness nowadays had improved a lot compared with that in 1990, so they would pay more attention to nutrition collocation rather than just solving the problem of food and clothing. Consequently, fruit had gradually become one of the main components of the diet. With the development of economy and medical technology, stroke mortality showed a significant decline with period. The increasing fruit consumption and decreasing stroke mortality together led to a decline in stroke mortality attributable to low fruit intake during 1990–2017. Although the average daily fruit consumption of population aged 18–44 years had increased significantly in China over the past 20 years, the overall level was still far below the standard recommended by WHO. Therefore, some effective and targeted measures should be taken to increase the population’s knowledge about the health benefits of fruit intake, thereby improving the current situation of low fruit intake and reducing the mortality risk of stroke caused by low fruit intake in China.

The cohort effect revealed various exposures of earlier life and risks in different birth cohorts, such as socioeconomic, behavioral, and environmental exposure (Sun et al., 2018). In the present study, the net cohort effect showed a significant downward trend; the earlier birth cohorts had suffered a higher mortality risk, while the more recent cohorts had experienced a lower risk, which was consistent with previous studies on stroke mortality and its attributable burden of high sodium intake. This overall declining trend was mainly due to the dramatic economic development and the improvement of medical and environmental conditions in China (Khellaf et al., 2010). Generally, a higher stroke mortality risk attributable to low fruit intake was found before 1949, which was attributed to the unstable socioeconomic development, poor nutrition intake and health services during their birth and childhood (Li et al., 2017). The RR remained stable between 1943 and 1947 and 1948 and 1952 birth cohorts for females, with males rising slightly, which might be due to the lagging impact of the World War II in China. In the first few years after World War II, the medical condition was stagnant, and fruits and foods were also very scarce; thereby the stroke mortality attributable to low fruit intake would be increased, so the 1943–1947 and 1948–1952 birth cohorts did not follow the decline pattern of the overall cohorts (Liczbinska et al., 2018). After the establishment of the People’s Republic of China in 1949, the stroke mortality risk due to low fruit intake in 1953–1957 and 1958–1962 birth cohorts showed a rapid downward trend, which was mainly because the medical conditions and health services had increased during this era, and the maternal and child health system was established and gradually improved during 1949–1966 (Guo et al., 2015). The stroke mortality risk due to low fruit intake in 1963–1967 birth cohort was higher than that in the earlier cohorts because this cohort had experienced the Great Cultural Revolution and baby boomer in China. The 1963–1967 cohort grew up in an unstable environment with poor nutrition and healthcare, and they were likely to be exposed to more risk factors of stroke, including low fruit intake. Therefore, this cohort suffered a higher mortality risk compared with the earlier cohorts (Wen et al., 2019). After the 1963–1967 cohort, the mortality risk showed a downward trend, and the change rates slowed down gradually. After the Reform and Opening Up in China, the mortality risk was at the lowest level in all cohorts, which was mainly due to the rapid economic development and medical improvement. In addition, people’s health awareness had gradually increased, and they paid more attention to chronic disease prevention. According to the National Institute for Nutrition and Health, the fruit consumption increased from 37.4 in 1980 to 49.2 in 1992, which was far below the recommended value by WHO, and the change rate was also not obvious (National Institute For Nutrition And Health, 2019). Additionally, the economy and healthcare were already at a higher level; therefore, the cohort effect after the reform had gradually stabilized.

Nevertheless, our study had some limitations. First, the results in our study depended on the GBD 2017, which integrated data from a variety of sources, including published and unpublished data. Low fruit intake in some data was not directly measured, so it may have some potential biases. However, there were very few available data on disease burden attributable to fruit intake in China, especially on long-term trends, so few studies have analyzed the chronic disease burden caused by low fruit intake over the past two decades. GBD 2017 evaluated the disease burden of low fruit intake from sex and age groups during 1990–2017, and many methods, including misclassification corrections and redistribution of garbage codes, were used to reduce bias, which was a more comprehensive study to analyze the long-term trends of disease burden attributable to low fruit intake than any previous global and national study (GBD 2017 Disease and Injury Incidence and Prevalence Collaborators, 2018). Second, our study lacked the analysis of comparison of stroke mortality attributable to low fruit intake between urban and rural areas because of unavailability of data, and our study was limited in populations in China, so more relevant studies in other countries and areas should be carried out in the future to reveal the characteristics of stroke mortality attributable to low fruit intake in different populations. Third, compared with the individualized study, the APC-IE framework concluded the results at population level, which would be affected by ecological fallacy.

In summary, our study estimated the long-term trends of stroke mortality attributable to low fruit intake by sex from 1990 to 2017. We found that the CMRs increased for males, while they decreased for females; the ASMRs for both sexes showed decreasing trends, and the crude and ASMRs for males were higher than those for females. By APC-IE analysis, stroke mortality attributable to low fruit intake increased with age from 25 to 79, then decreased in the 80–84 age group for both sexes. The independent period and cohort effects progressively decreased during the entire period for both sexes, with a faster decrease for females than for males. Males and elder groups were the high-risk population for stroke mortality caused by low fruit intake. Although the mortality risk showed a decreasing trend, the fruit intake was still low for people in China. Therefore, effective strategies and global awareness are essential to improve the current situation of low fruit intake, thereby preventing and reducing the stroke mortality risk caused by low fruit intake in China.

## Data Availability Statement

All datasets presented in this study are included in the article/Supplementary Material.

## Ethics Statement

Ethical approval was not required because of the public and anonymous characteristics of macro data.

## Author Contributions

LL, JJ, and YJ conceptualized and designed the study. LL, CY, MZ, YW, and JJ collected and assembled the data. LL, JJ, and QL analyzed and interpreted the data. LL, JJ, MZ, and YW drafted the manuscript. CY, YJ, QL, JJ, and LL revised the manuscript critically for important intellectual content. All authors contributed to the article and approved the submitted version.

## Conflict of Interest

The authors declare that the research was conducted in the absence of any commercial or financial relationships that could be construed as a potential conflict of interest.
