# Supplementary material for: Stroke Mortality Attributable to Low Fruit Intake in China: A Joinpoint and Age-Period-Cohort Analysis
Source: Front Neurosci. 2020 Dec 1;14:552113. doi: 10.3389/fnins.2020.552113 (PMC7736244; doi:10.3389/fnins.2020.552113)
Supplement: Supplementary file 1 [file Table_1.DOCX]

Table S1 The age, period and cohort effects of stroke mortality attributable to low in fruit intake by sex in China .

| Factors | Males | | |  | Females | | |
| --- | --- | --- | --- | --- | --- | --- | --- |
|  | Coef | 95% CI | SE |  | Coef | 95% CI | SE |
| Intercept | 3.83 | (3.73,3.92) | 0.05 |  | 3.24 | (3.09,3.40) | 0.08 |
| Age |  |  |  |  |  |  |  |
| 25-29 | -1.85 | (-2.23, -1.48) | 0.19 |  | -1.83 | (-2.32, -1.33) | 0.25 |
| 30-34 | -1.48 | (-1.77, -1.19) | 0.15 |  | -1.60 | (-2.01, -1.19) | 0.21 |
| 35-39 | -1.32 | (-1.58, -1.05) | 0.13 |  | -1.46 | (-1.83, -1.09) | 0.19 |
| 40-44 | -0.74 | (-0.94, -0.54) | 0.10 |  | -0.92 | (-1.20, -0.64) | 0.14 |
| 45-49 | -0.43 | (-0.6-, -0.26) | 0.09 |  | -0.44 | (-0.66, -0.23) | 0.11 |
| 50-54 | -0.08 | (-0.22, 0.06) | 0.07 |  | -0.08 | (-0.25, 0.10) | 0.09 |
| 55-59 | 0.18 | (0.06, 0.30) | 0.06 |  | 0.15 | (0.01, 0.30) | 0.08 |
| 60-64 | 0.61 | (0.51, 0.70) | 0.05 |  | 0.57 | (0.45, 0.69) | 0.06 |
| 65-69 | 0.93 | (0.85, 1.01) | 0.04 |  | 0.94 | (0.83, 1.04) | 0.05 |
| 70-74 | 1.33 | (1.25, 1.40) | 0.04 |  | 1.38 | (1.28, 1.49) | 0.06 |
| 75-79 | 1.52 | (1.44, 1.60) | 0.04 |  | 1.68 | (1.55, 1.81) | 0.06 |
| 80-84 | 1.34 | (1.24, 1.44) | 0.05 |  | 1.60 | (1.44, 1.76) | 0.08 |
| Period |  |  |  |  |  |  |  |
| 1992 | 0.01 | (-0.07, 0.09) | 0.04 |  | 0.25 | (0.14, 0.36) | 0.05 |
| 1997 | 0.00 | (-0.06, 0.06) | 0.03 |  | 0.14 | (0.06, 0.22) | 0.04 |
| 2002 | 0.06 | (0.01, 0.11) | 0.03 |  | 0.09 | (0.03, 0.15) | 0.03 |
| 2007 | -0.04 | (-0.09, 0.02) | 0.03 |  | -0.12 | (-0.19, -0.06) | 0.03 |
| 2012 | -0.01 | (-0.08, 0.05) | 0.03 |  | -0.15 | (-0.24, -0.06) | 0.04 |
| 2017 | -0.02 | (-0.10, 0.06) | 0.04 |  | -0.21 | (-0.33, -0.10) | 0.06 |
| Cohort |  |  |  |  |  |  |  |
| 1908-1912 | 0.77 | (0.61, 0.93) | 0.08 |  | 0.65 | (0.44, 0.87) | 0.11 |
| 1913-1917 | 0.71 | (0.59, 0.83) | 0.06 |  | 0.68 | (0.51, 0.85) | 0.09 |
| 1918-1922 | 0.67 | (0.57, 0.77) | 0.05 |  | 0.67 | (0.53, 0.82) | 0.07 |
| 1923-1927 | 0.63 | (0.54, 0.72) | 0.05 |  | 0.66 | (0.53, 0.79) | 0.07 |
| 1928-1932 | 0.50 | (0.41, 0.58) | 0.04 |  | 0.6 | (0.47, 0.73) | 0.07 |
| 1933-1937 | 0.37 | (0.28, 0.47) | 0.05 |  | 0.51 | (0.37, 0.65) | 0.07 |
| 1938-1942 | 0.22 | (0.11, 0.33) | 0.06 |  | 0.38 | (0.22, 0.54) | 0.08 |
| 1943-1947 | 0.11 | (-0.02 ,0.25) | 0.07 |  | 0.3 | (0.11, 0.49) | 0.10 |
| 1948-1952 | 0.12 | (-0.03, 0.28) | 0.08 |  | 0.29 | (0.06, 0.51) | 0.11 |
| 1953-1957 | -0.03 | (-0.22, 0.15) | 0.09 |  | 0.07 | (-0.20, 0.34) | 0.14 |
| 1958-1962 | -0.25 | (-0.47, -0.03) | 0.11 |  | -0.21 | (-0.53, 0.11) | 0.16 |
| 1963-1967 | -0.18 | (-0.41, 0.05) | 0.12 |  | -0.17 | (-0.52, 0.17) | 0.18 |
| 1968-1972 | -0.40 | (-0.69, -0.11) | 0.15 |  | -0.41 | (-0.84, 0.02) | 0.22 |
| 1973-1977 | -0.72 | (-1.10, -0.34) | 0.20 |  | -0.79 | (-1.39, -0.19) | 0.31 |
| 1978-1982 | -0.82 | (-1.32, -0.32) | 0.26 |  | -1.01 | (-1.83, -0.19) | 0.42 |
| 1983-1987 | -0.83 | (-1.45, -0.21) | 0.32 |  | -1.06 | (-2.09, -0.02) | 0.53 |
| 1988-1992 | -0.87 | (-1.99, 0.26) | 0.58 |  | -1.17 | (-3.04, 0.70) | 0.95 |
| Deviance | 11.58 | | |  | 5.30 | | |
| AIC | 6.74 | | |  | 6.14 | | |
| BIC | -159.49 | | |  | -165.77 | | |

Note: Coef: Coefﬁcient; SE: Standard error; CI: Conﬁdence interval; AIC: Akaike Information Criterions; BIC: Bayesian Information Criterions.
